# A Collaborative Brain-Computer Interface Framework for Enhancing Group Detection Performance of Dynamic Visual Targets

**DOI:** 10.1155/2022/4752450

**Published:** 2022-01-18

**Authors:** Xiyu Song, Ying Zeng, Li Tong, Jun Shu, Qiang Yang, Jian Kou, Minghua Sun, Bin Yan

**Affiliations:** ^1^Henan Key Laboratory of Imaging and Intelligent Processing, PLA Strategic Support Force Information Engineering University, Zhengzhou, China; ^2^The Clinical Hospital of Chengdu Brain Science Institute, MOE Key Lab for Neuro Information, University of Electronic Science and Technology of China, Chengdu, China; ^3^Xi'an Satellite Control Center, Hangzhou, China; ^4^PLA 32317 Force, Wulumuqi, China; ^5^Department of Radiology, Henan Provincial People's Hospital, Department of Radiology, Central China Fuwai Hospital, Zhengzhou University, Zhengzhou, China

## Abstract

The superiority of collaborative brain-computer interface (cBCI) in performance enhancement makes it an effective way to break through the performance bottleneck of the BCI-based dynamic visual target detection. However, the existing cBCIs focus on multi-mind information fusion with a static and unidirectional mode, lacking the information interaction and learning guidance among multiple agents. Here, we propose a novel cBCI framework to enhance the group detection performance of dynamic visual targets. Specifically, a mutual learning domain adaptation network (MLDANet) with information interaction, dynamic learning, and individual transferring abilities is developed as the core of the cBCI framework. MLDANet takes P3-sSDA network as individual network unit, introduces mutual learning strategy, and establishes a dynamic interactive learning mechanism between individual networks and collaborative decision-making at the neural decision level. The results indicate that the proposed MLDANet-cBCI framework can achieve the best group detection performance, and the mutual learning strategy can improve the detection ability of individual networks. In MLDANet-cBCI, the F1 scores of collaborative detection and individual network are 0.12 and 0.19 higher than those in the multi-classifier cBCI, respectively, when three minds collaborate. Thus, the proposed framework breaks through the traditional multi-mind collaborative mode and exhibits a superior group detection performance of dynamic visual targets, which is also of great significance for the practical application of multi-mind collaboration.

## 1. Introduction

Brain-computer interface (BCI) technology aims to build an interaction bridge between human and computer and provide a new technical means for the brain to control and monitor the external environment. Advanced BCI technology can not only help to improve the movement abilities of patients with physical disorders [1, 2], but also enhance such abilities of healthy people [3–7]. Affected by changes in the surrounding environment and in the psychological factors of users, a single-mind BCI shows limited performance and thus is hard to translate into practical application. Multi-mind collaborative brain-computer interfaces (cBCIs) have special advantages in enhancing the group detection performance. Multi-mind BCIs are equivalent to multiple information processing systems, which show higher group decision-making performance and stronger robustness. In addition, multi-mind collaborative work is more conducive to the future development of human-computer interaction socialization. P300-based BCIs broaden the BCI's practical application. The classical visual speller and target detection are based on P300 identification [8, 9]. For the task of dynamic target detection, the dynamics of video background, the uncertainty of distractors, and the jitter of detection latency increase the detection difficulty, resulting in the limitations of single-mind BCI [10, 11]. The cBCI can be considered as a good strategy to solve the problem, which will contribute to improving the stability and accuracy of comprehensive discrimination [12, 13]. Therefore, building a novel cBCI framework to highlight the performance advantages of multi-mind enhancement has become the research focus to improve the performance of dynamic visual target detection.

The cBCIs are praised as one of the most promising applications in human augmentation [14–16]. The group decision-making capability can be improved by integrating the multi-mind information and optimizing collaborative strategies [17, 18]. For the task of target detection, the multi-mind collaborative information integration mainly includes three levels: signal-level fusion, feature-level fusion, and decision-level fusion. Signal-level fusion is the simplest way to improve the signal-to-noise ratio (SNR) of EEG signals, where multi-participant EEG signals are averaged and input to a classifier. Feature-level fusion is the classification of the averaged or concatenated features from multi-participant EEG signals. Both signal-level fusion and feature-level fusion belong to single-classifier cBCI (SC-cBCI). Decision fusion merges the multiple classifiers' decision-making results into the final decision. Each classifier corresponds to one participant. Thus, decision fusion is also known as multi-classifier cBCI (MC-cBCI). The specific decision emergence strategies include averaged decision, weighted decision, and majority voting on the decision probability layer. The cBCI has attracted scholars' interest in target detection. To explore the best fusion level, Matran-Fernandez et al. preliminarily verified that the decision fusion in the cBCI performs better than any single-mind BCI (sBCI) for the single-trial P300 detection [19–21]. The relevant studies indicated that the decision-level fusion performs better than signal-level fusion and feature-level fusion in the cBCI [22]. To explore the best decision emergence strategies, Cecotti et al. [23, 24] found that the averaged decision performs better than weighted decision and voting strategy; Davide et al. [15, 25–27], Yuan et al. [28, 29], and Jiang et al. [30] trained individual decision weights through least angle regression (LARS) method, two-layer SVM, and a combination of SVM and LDA classifiers to improve group detection performance. Since the above findings are inconsistent, the selection of decision fusion strategies relies on the specific experimental tasks. To introduce the information interaction among multiple minds, Davide et al. [16] studied the impact of individual behavior decision sharing on collaborative behavior decision-making and found that information interaction in the experimental process will lead to the decline of behavior-level collaborative decision-making performance. To fuse more information and improve the group detection performance, Zhang et al. [31] proposed a dual brain collaborative target detection model, which integrates data fusion and feature fusion to ensure that important information is not missed; Eckstein et al. [32] explored and compared the impact of the user number on the collaborative decision performance and found that the best collaborative detection performance generally requires 5∼10 users. These studies provide technical reference and theoretical support for the design of multi-mind collaborative experimental paradigm and the development of cBCI framework.

Studies on multi-mind collaborative target detection have achieved remarkable results. However, some concerning issues remain. Firstly, in the current cBCIs, the computing models are static and unidirectional [18, 32–34] only be known as “multi-mind fusion” rather than “multi-mind collaboration.” Real collaboration should involve multi-mind information interaction, which is a dynamic learning process. Given the higher error rate caused by the individual communication in the experimental process [16], relative to behavior level, the information interaction can be established at the neural decision layer. Thus, a dynamic interactive cBCI framework at neural decision layer could be considered to improve the performance. Secondly, previous studies required participants to increase the preparation time to collect labeled signals for an individual-specific computing model [26, 27, 34]. For the dynamic visual target detection, unsupervised domain adaptation networks, P3-MSDA and P3-sSDA, have been developed as an individual-general network with reliable performance in previous studies [35]. Therefore, it is necessary to develop a novel cBCI framework with information interaction, dynamic learning, and individual transferring abilities to enhance the group detection performance video targets.

In this study, we designed a novel multi-mind cBCI framework based on a mutual learning domain adaptation network (MLDANet), aiming at enhancing the group detection performance of dynamic visual targets. In the framework, a multi-mind synchronous cBCI experimental paradigm for UAV-video vehicle detection is designed; MLDANet is established where the P3-sSDA network is used as the individual network unit. This work made the following contributions.

1. The MLDANet-cBCI framework was proposed for achieving better group detection performance. In particular, MLDANet establishes the information interaction and dynamic learning mechanism between individual networks and collaborative decision-making by introducing the mutual learning strategy at the neural decision layer.
2. In the MLDANet-cBCI framework, the mutual learning strategy can effectively improve the individual network capability.

## 2. Materials and Methods

**Figure 1:**
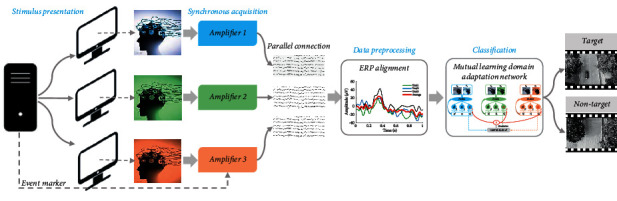
Collaborative BCI framework for detecting dynamic visual targets.

This collaborative brain-computer interface (cBCI) framework is designed to detect dynamic visual targets, as shown in Figure 1. The framework consists of four modules, which are stimulus presentation, synchronous acquisition, data preprocessing, and classification. The stimulus presentation module synchronously shows unmanned aerial vehicle (UAV) videos to all participants to detect the vehicles from these videos. The synchronous acquisition module collects multi-mind EEG signals with time synchronization. The data preprocessing module aims to obtain artifact-free and filtered EEG epochs for target and nontarget trials. The classification module is the core of the cBCI framework, where a mutual learning domain adaptation network (MLDANet) is proposed to improve the group detection performance.

### 2.1. Stimulus Presentation

**Figure 2:**
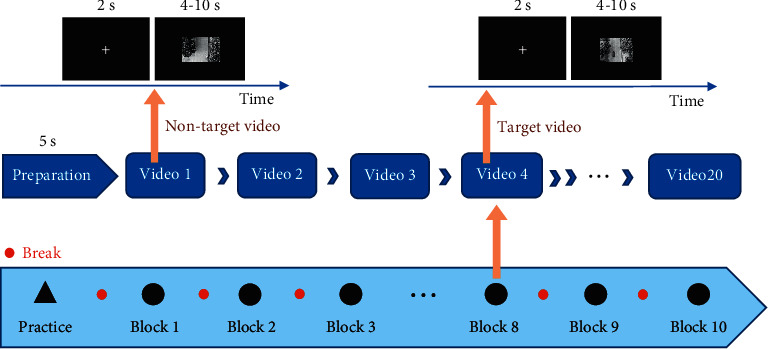
Experimental paradigm for vehicle detection in UAV video.

The experimental paradigm for vehicle detection from UAV videos, reported in our previous study [5], is depicted in Figure 2. The UAV videos recorded traffic conditions while flying along campus streets. A series of video clips were segmented from original videos to construct a stimulus library. One hundred video clips with vehicles (one vehicle per video) and 100 video clips without vehicles were, respectively, regarded as target videos and nontarget videos. In this experiment, the total duration time of videos is about 28 minutes. To alleviate the vision load, we divided all the video clips to 10 blocks and set break time between blocks. In each block, 10 target videos and 10 nontarget videos were randomly presented to the participants in each block. The length of the video clip varied from 4 s to 10 s, and there were 2 s “+” before each video to help participants focus their attention. For each target video, the vehicle could enter the visual field from any direction at any time 1 s after the video stimulus was presented. To overcome the influence of video color and eye movement on visual perception, the video clips (1920 × 1080 px^2^) were transformed into black and white and reduced to 40% (768 × 432 px^2^) on the screen center against black background. In particular, the break duration totally depended on the subjects for sufficient relax. On average, the experimental duration time of 10 blocks (including break duration) was around 50 minutes for one participant.

### 2.2. Synchronous Acquisition

A total of 89 healthy college participants volunteered for this study, with a median age of 25 years (right-handed), all of whom reported normal or corrected-to-normal vision and presented no neurological problems. They all signed the informed consent form before the experiment. All tests involving human participants were approved by the Ethics Committee of Henan Province People's Hospital.

In this study, the EEG signals were collected by the g.USBamp (g.tec, Austria) EEG recording with 16 electrodes. The electrode distribution followed the international 10–20 electrode location. The EEG online sampling rate was 600 Hz with band-pass filtering at 0.01–100 Hz and notch filtering at 50 Hz.

**Figure 3:**
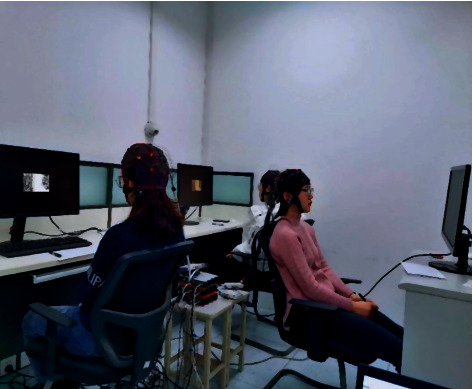
The EEG acquisition environment for the multi-mind synchronous experiment.

The study was comprised of two parts: single-mind experiment and multi-mind experiment. 29 participants were recruited for the single-mind experiment, and each time only a single participant was invited to perform the detection task. The EEG signals collected from the single-mind experiment were used as the training set. In the multi-mind experiment, 20 groups (3 participants in each group) were recruited to perform the detection task together. The acquisition environment for the multi-mind synchronous experiment is shown in Figure 3. The same stimulus materials were simultaneously displayed on three copied displays. There was no communication between the three participants during the experiment. Meanwhile, EEG signals from 3 participants were synchronously collected by three parallel EEG amplifiers and recorded by recording software (g.Recorder). The recording software arranged these 16-channel signals. Channels 1∼16, channels 17∼32, and channels 33∼48 were, respectively, from participant 1, participant 2, and participant 3. In this manner, the synchronization of time, space, and surrounding environment ensured that the influence of external factors on each participant was identical. All the collected data will be made available to peers for any relevant future work.

### 2.3. Data Preprocessing

The parameter settings of data preprocessing were consistent between single-mind and multi-mind experiments. Firstly, using the fast ICA algorithm and EEGLAB toolbox, the electrooculogram was removed from the original signals. Next, data were filtered to 0.1–10 Hz and downsampled to 100 Hz. Then, target segments and nontarget segments were, respectively, extracted from target video-induced and nontarget video-induced EEG signals. The target trials were segmented starting from target onset time. One target video can only induce one target trial, and nontarget trials were segmented from nontarget video-induced signals without overlapping, where one nontarget video can contain several nontarget trials. Thus, 100 target trials from 100 target videos and 521 nontarget trials from 100 nontarget videos were extracted. The signals for each trial were 1500 ms. The size of the single-trial sample was 16 × 150 (channels × time sample points).

Since a domain adaptation network was applied to predict the detection performance. Single-mind signals and multi-mind signals were, respectively, used to construct the source domain and the target domain.

#### 2.3.1. Single-Mind Signals (Source Domain)

Single-mind signals from 29 participants were employed to construct the training set. In our previous studies [35], individuals with strong P3 responses by P3 map-clustering method as source domains help to achieve better detection performance. Here, the P3 map-clustering method was applied to select excellent individuals as the source domain for better detection performance. Due to the serious time jitter of P300 latency for the video-induced EEG signals, before using the P3 map-clustering method, individual P3 maps had to be extracted by the event-related potential (ERP) alignment method [11]. The principle of this method is to reduce the spatial dimension of the single-trial signal to construct a one-dimensional target ERP template by the common spatial patterns (CSP) [6, 36], and match all the one-dimensional time series with the ERP template to obtain the aligned P300 signals. Using the constructed 1000 ms ERP template, the size of the aligned trials was determined as 16 × 100 (channels × time sample points). The brain topographic map at a peak time of the P300 component was extracted as an individual P3 map. Here, 29 individual P3 maps were obtained from 29 participants. Using a *K-means* distance-clustering method with two clustering centers, 29 maps were clustered into two groups with strong P3 maps and weak P3 maps [35]. Subsequently, the individuals in the strong P3 map group were considered to act as the source domain.

#### 2.3.2. Multi-Mind Signals (Target Domain)

Multi-mind signals were used as the testing set. Using the ERP template constructed from the source domain, there were 621 aligned trials in total (100 target trials and 521 nontarget trials) available for each individual, which constituted an imbalanced dataset. The size of the single-trial sample was 16 × 100 (channels × time sample points). The validity of the single-trial signals was tested to screen some samples as target domain for domain adaptation network. The threshold value method was adopted for the sample screening. The single-trial signals with maximum amplitude values within ±120 *μ*V were regarded as valid signals; otherwise, they were regarded as invalid signals. Thus, each single-trial EEG signal corresponded to two labels (category label and validity label) for each participant.

### 2.4. Classification Model

**Figure 4:**
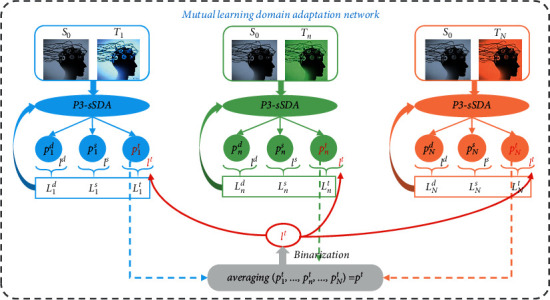
The architecture of MLDANet for group detection.

Aimed at enhancing the performance of group detection of dynamic visual targets, MLDANet is proposed as shown in Figure 4. The core of MLDANet is to establish the mechanisms of multi-mind information interaction and dynamic learning at the neural decision layer. In the MLDANet, the collaborative decision-making guides the individual network to re-decision-making for the enhancement of collaborative decision-making performance.

**Figure 5:**
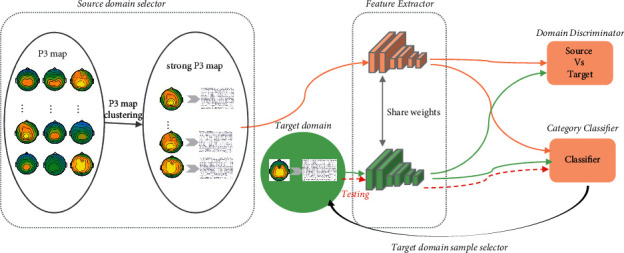
P3-sSDA network architecture.

In the proposed framework, an unsupervised single-source domain adaptation network with strong P3 map individuals as the source domain for dynamic visual target detection (P3-sSDA network) is used as the individual network unit [35]. The P3-sSDA network is an individual-generalized model with good performance in EEG-based dynamic visual target detection, as shown in Figure 5. P3-sSDA consists of five parts: source domain selector, feature extractor, domain discriminator, category classifier, and target domain sample selector. In P3-sSDA, a P3 map-clustering method selected the individuals with strong P3 maps as one source domain. Feature extractor extracts the EEG features from video targets-induced EEG signals. Domain discriminator performs the adversarial domain adaptation to eliminate individual differences. Category classifier classifies the EEG features to distinguish target samples and nontarget samples. The testing samples are ranked according to the probability value predicted as target samples. Target domain sample selector selects the samples most like the target samples from testing samples as the target domain samples for the imbalanced data classification. The proportion of samples selected is 80%. In this study, the training individuals and testing individuals were completely independent. Thus, the P3-sSDA network was suitable for establishing individual-generalized cBCI frameworks for dynamic visual target detection. The detailed network architecture was given in our previous work [35].

In the MLDANet, there were *N* target domain individuals from one group with *N* P3-sSDA networks, which were used as classifiers that synchronously worked on predicting the binary classification probability of single-trial signals. Collaborative decision-making was achieved by the decision-making fusion. The mutual learning strategy was introduced between *N* P3-sSDA networks and collaborative decision-making. Data from the common source domain and different target domains were, respectively, denoted as *S*_0_, *T*_1_, *T*_2_,…, and *T*_*N*_, where *N* P3-sSDA networks shared the same source samples and used *N* different target domain samples. For a single P3-sSDA network, the input sample number of source domain or target domain was named as batch size *m*. For the *n*-th target domain individual, *m* source domain samples *x*_1_^*s*^, *x*_2_^*s*^,…, *x*_*m*_^*s*^ and *m* target domain samples *x*_1_^*t*_*n*_^, *x*_2_^*t*_*n*_^,…, *x*_*m*_^*t*_*n*_^ were input into the *n*-th P3-sSDA network in each batch. Importantly, target domain samples from *N* target domain individuals were synchronously recorded for the same stimulus scene. Thus, the prediction category labels of the target domain could be shared among *N* P3-sSDA networks, which was crucial to achieve the information interaction. For each iteration, the P3-sSDA network could output the domain discriminant probability between source domain samples and target domain samples, the category prediction probability of source domain samples, and the category prediction probability of target domain samples. These probabilities were, respectively, denoted as *p*_*n*_^*d*^, *p*_*n*_^*s*^, and *p*_*n*_^*t*^ for the *n*-th P3-sSDA network. Since *N* individuals synchronously received the same stimuli information, the prediction probability, *p*_1_^*t*^, *p*_2_^*t*^,…, and *p*_*N*_^*t*^, could reflect the discrimination level for the same information from different individuals. In the process of domain adaptation, the category labels of the source domain *l*^*s*^ and the domain labels *l*^*d*^ were available; hence, the category loss of the source domain was given as

(1) $$\begin{array}{c} L_{\text{class}}^{s_{t_{n}}}=-\sum_{k=1}^{m}\left(l_{k}^{s}\log\;p_{n}^{s,k}\right), \end{array}$$

and the domain loss between the source domain and the *n*-th target domain, *L*_*a*  *dv*_^*s*,*t*_*n*_^, could be obtained for the *n*-th target domain:

(2) $$\begin{array}{c} L_{a\;dv}^{s,t_{n}}=-\sum_{k=1}^{m}\left(l_{k}^{d,s}\log\;p_{n}^{d,s,k}+l_{k}^{d,t}\log\;p_{n}^{d,t,k}\right). \end{array}$$

Nevertheless, the category loss of the target domain was unknown due to the lack of category labels of the target domain *l*^*t*^, which would be estimated in each iteration. By averaging the individual predictions, the integrated prediction probability *p*^*t*^ was binarized as *l*^*t*^:

(3) $$\begin{array}{c} l^{t}=\text{binary}\left(p^{t}\right)=\text{binary}\left(\text{averaging}\left(p_{1}^{t},p_{2}^{t},\ldots ,p_{N}^{t}\right)\right), \end{array}$$

which was viewed as the prediction results of the multiple minds. Moreover, the integrated prediction label *l*^*t*^ was used as the pseudo-label and synchronously fed back to *N* P3-sSDA networks to calculate the category losses of target domain {*L*_class_^*t*_*n*_^}_*n*=1_^*N*^.

(4) $$\begin{array}{c} L_{\text{class}}^{t_{n}}=-\sum_{k=1}^{m}\left(l_{k}^{t}\log\;p_{n}^{t,k}\right). \end{array}$$

Thus, the category and discrimination losses could be calculated. For the MLDANet with *N* P3-sSDA network, the entire adversarial learning problem could be described as follows:

(5) $$\begin{array}{c} \min\sum_{n=1}^{N}\left(a\times L_{a\;dv}^{s,t_{n}}+\gamma \times L_{\text{class}}^{s_{t_{n}}}+\beta \times L_{\text{class}}^{t_{n}}\right), \end{array}$$

where *L*_*a*  *dv*_^*s*,*t*_*n*_^, *L*_class_^*s*_*t*_*n*__^, and *L*_class_^*t*_*n*_^ denote the domain discrimination loss between the source domain and the *n*-th target domain, the category loss of the source domain when adapted to the *n*-th target domain, and the category loss of the *n*-th target domain, respectively; *α*, *γ*, and *β* are hyperparameters which, respectively, denote discrimination loss weight, category loss weight of source domain, and category loss weight of target domain.

The mechanism of information interaction among multi-mind signals was established through information integration and feedback. In the MLDANet framework, each individual network not only receives the supervision from individual network labels, *l*^*d*^ and *l*^*s*^, but also refers to the collaborative label *l*^*t*^, which is calculated from all individual networks. By the backpropagation of collaborative label *l*^*t*^, all the individual networks can learn from each other and make common progress. This process can help small network training to be more powerful. Different from classical cBCI with once collaborative decision-making, with the iteration and updating of network parameters, the dynamic learning ability of the individual network was established in the MLDANet. Thus, the single P3-sSDA network (single individual) could learn from the source domain, target domain, and collaborative decision-making (group). After the training process of DA, the online testing can be conducted as the procedure of red dotted line in Figure 5. The new testing samples can directly be tested by feature extractor and category classifier.

## 3. Results

### 3.1. Source Domain Individuals

**Figure 6:**
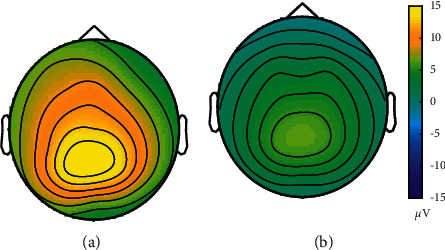
The averaged P3 map of two groups: (a) strong P3 map; (b) weak P3 map.

**Figure 7:**
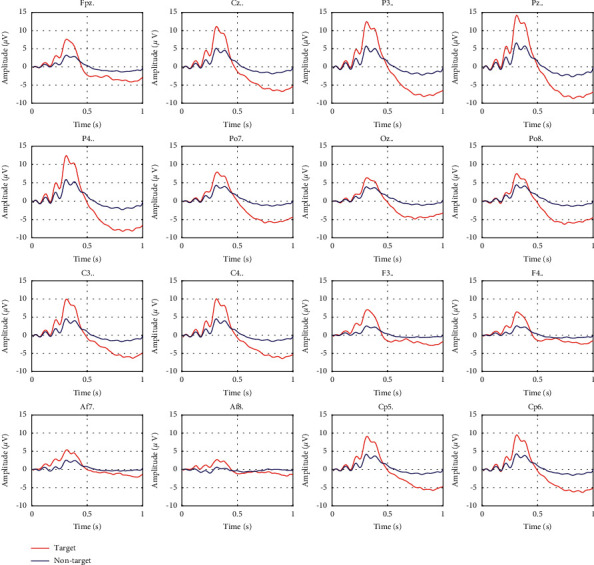
The averaged ERP responses of source domain individuals.

A total of 29 individual P3 maps were clustered into a strong P3 group {*sub*6, *sub*11, *sub*12, *sub*13, *sub*14, *sub*16, *sub*17, *sub*19, *sub*20, *sub*21, *sub*23, *sub*26, *sub*28} and a weak P3 group {*sub*1, *sub*2, *sub*3, *sub*4, *sub*5, *sub*7, *sub*8, *sub*9, *sub*10, *sub*15, *sub*18, *sub*22, *sub*24, *sub*25, *sub*27, *sub*29}. The topographies of the strong P3 group and the weak P3 group at the P3 peak value are presented in Figure 6, where the parieto-occipital region is closely related to P3 responses. Since studies indicated that individuals with strong P3 maps are more suitable as the source domain, we selected all individuals from the strong P3 group as the source domain. The averaged ERP responses of source domain individuals (13 individuals from the strong P3 group) are shown in Figure 7.

### 3.2. Detection Performance

**Figure 8:**
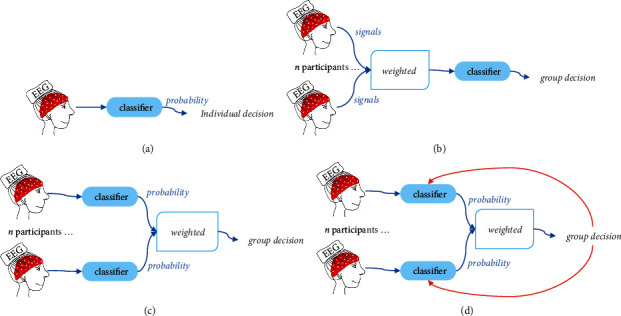
Summary of different BCI frameworks: (a) sBCI framework; (b) SC-cBCI framework; (c) MC-cBCI framework; (d) MLDANet-cBCI framework.

In this work, we compared the detection performances of four BCI frameworks, namely, the single-mind BCI (sBCI), the single-classifier cBCI (SC-cBCI), the multi-classifier cBCI (MC-cBCI), and the proposed MLDANet-cBCI framework. The summary of these BCI frameworks is shown in Figure 8. One individual participates in the sBCI framework, where the results are averaged from 60 individuals. The SC-cBCI, MC-cBCI, and MLDANet-cBCI frameworks belong to the multi-mind framework, where results are the averaged value of 20 groups. The SC-cBCI framework involves signal-level fusion, while the MC-cBCI framework and the MLDANet-cBCI framework perform decision-level fusion. In particular, the MLDANet-cBCI framework introduces the mutual learning strategy, and the decision-making is dynamic and interactive.

The optimal parameter values are shown in Table 1, with all frameworks trained on an NVIDIA TITAN RTX GPU in the PyTorch platform. We fit the model using the Adam optimizer with cross-entropy function. The weighted coefficients among the three participants were (1,1,1), where the collaborative decision-making was obtained by averaging three decision-making probabilities.

**Figure 9:**
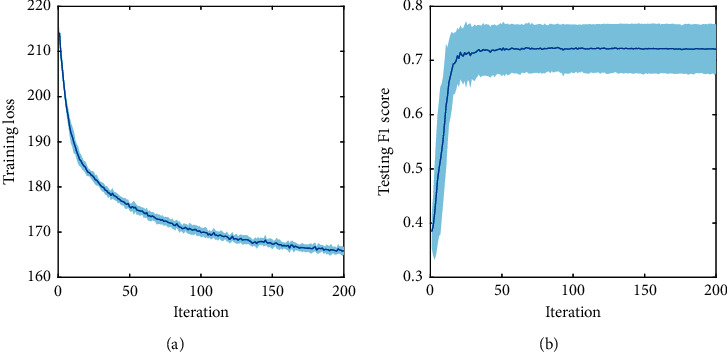
The model convergence in the MLDANet-cBCI framework: (a) the convergence of training loss; (b) the convergence of F1 score.

The detection performances of different BCI frameworks are shown in Table 2, namely, classification accuracy, hit rate, false alarm rate, and F1 score. Here, F1 score is viewed as the main performance evaluation criterion due to the imbalanced classification. The significance level by analysis of variance (ANOVA) was performed between MLDANet-cBCI and other cBCI frameworks. The results indicated that cBCI frameworks outperform the sBCI framework. In cBCI frameworks, decision-level fusion (MC-cBCI and MLDANet-cBCI) outperformed signal-level fusion (SC-cBCI). The MLDANet-cBCI with mutual learning strategy performed the best. Compared with the MC-cBCI framework, the F1 score of MLDANet-cBCI framework improved by 0.12, which highlighted the superiority of the MLDANet-cBCI framework. Relatively, both hit rate and false alarm rate of MLDANet-cBCI framework were lower than those of SC-cBCI framework, which illustrates that MLDANet generates a higher decision threshold. The convergence of training loss and testing F1 score in the MLDANet-cBCI framework from 20 groups is shown in Figure 9. When the iterations exceed 150 rounds, the training loss was unchanged; when the iterations exceeded 50 rounds, the detection performance became stable.

## 4. Discussion

### 4.1. Effects of Mutual Learning Strategy on Individual Network Capability

It was expected that the mutual learning strategy would improve the individual network capability of the MLDANet-cBCI framework. Here, the individual detection performance for 20 groups in the MC-cBCI and MLDANet-cBCI frameworks was shown (Table 3). The results indicated that the individual F1 score was 0.66 in the MLDANet-cBCI framework, which is significantly higher than that in the MC-cBCI framework (*p* < 0.01), and could even exceed the group performance in the SC-cBCI framework (0.59) and the MC-cBCI framework (0.61) (Table 2). This finding further confirmed that the proposed mutual learning strategy improves the information interaction and dynamic learning capability of individual networks in the MLDANet-cBCI framework, where individual networks with so-called poor classification performance can also be cultivated to reach the expert level. This shows that the so-called poor detection performance does not necessarily mean that the data is unreliable; it is likely that the features are not obvious or difficult to extract, and the network training mode also has a great impact on the performance. With the mutual learning strategy, the MLDANet-cBCI framework effectively breaks through the bottleneck of traditional cBCI detection.

### 4.2. Effects of Number of Individuals in the Source Domain on Detection Performance

**Figure 10:**
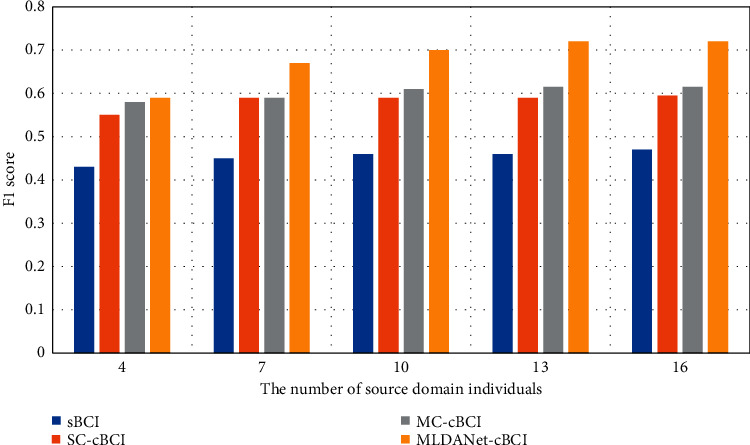
Detection performances with different number of individuals in the source domain.

In order to achieve a better detection performance, the optimal number of source domain individuals was evaluated on different BCI frameworks. According to the previous work, source domain individuals with stronger P3 maps contribute to better detection performance [35]. When there were few individuals in the source domain, individuals with stronger P3 maps were preferentially selected as the source domain for better performance. In order to observe the change trend of detection performance with different number of individuals in the source domain and simplify the calculation, source domain individuals were sorted in descending order according to P3 maps of 29 participants. In addition, the first 4, 7, 10, 13, and 16 individuals were used to construct the source domain, as shown in Figure 10. The results indicated that around 13∼16 source domain individuals could contribute to the improved performance for the multi-mind EEG signals in this paradigm. Furthermore, the MLDANet-cBCI framework always showed the best performance. Notably, the proposed MLDANet-cBCI framework was particularly sensitive to the number of source domain individuals, where the F1 score improved by 0.14 with their number varying from 4 to 13. Thus, the superiority of MLDANet was based on a sufficient number of source domain individuals.

## 5. Conclusions

In the present work, we developed a multi-mind cBCI framework to enhance the group detection performance of dynamic visual targets. In this framework, a mutual learning domain adaptation network (MLDANet) was proposed to establish the mechanisms of information interaction and dynamic learning between individual network units and collaborative decision-making. By a mutual learning strategy, the collaborative decision-making could guide the re-decision-making process of the individual network for better and more robust detection performance. The results indicated that the proposed MLDANet-cBCI framework outperforms the other cBCI frameworks with the highest F1 score, 0.73, and classification accuracy, 0.91, when three participants collaborate. The mutual learning strategy can effectively improve the individual network capability. Therefore, the proposed cBCI framework provides a novel multi-mind collaborative mode for the improvement of collaborative work performance, which is of great significance for the progression of research on human augmentation.

## Figures and Tables

**Table 1:** Network parameter settings.

Parameters	Value		
	sBCI/SC-cBCI	MC-cBCI	ML-cBCI
P3-sSDA network	1	3	3
Batch size	40	40	40
Learning rate	0.0003	0.0003	0.0003
Epoch	100	100	100
*α*	0.2	0.2	0.4
*γ*	0.8	0.8	0.8
*β*	0.2	0.2	0.2

**Table 2:** Detection performances on different BCI frameworks (^*∗∗*^*p* < 0.01).

BCI frameworks	Accuracy	Hit rate	False alarm rate	F1 score
sBCI	0.77	0.63	0.20	0.47(^*∗∗*^)
SC-cBCI	0.82	0.80	0.18	0.59(^*∗∗*^)
MC-cBCI	0.86	0.69	0.11	0.61(^*∗∗*^)
MLDANet-cBCI	0.91	0.72	0.05	0.73

**Table 3:** Detection performance of the individual network in the MC-cBCI and MLDANet-cBCI frameworks.

Groups	Individual F1 score in the MC-cBCI framework			Individual F1 score in the MLDANet-cBCI framework		
	Participant 1	Participant 2	Participant 3	Participant 1	Participant 2	Participant 3
Group 1	0.55	0.37	0.53	0.71	0.63	0.65
Group 2	0.43	0.48	0.37	0.56	0.61	0.53
Group 3	0.42	0.54	0.44	0.33	0.60	0.71
Group 4	0.40	0.49	0.52	0.63	0.69	0.67
Group 5	0.43	0.56	0.45	0.71	0.76	0.76
Group 6	0.45	0.48	0.50	0.70	0.72	0.74
Group 7	0.48	0.41	0.44	0.55	0.55	0.60
Group 8	0.49	0.40	0.50	0.69	0.66	0.68
Group 9	0.38	0.55	0.47	0.59	0.74	0.65
Group 10	0.33	0.53	0.57	0.45	0.78	0.74
Group 11	0.32	0.60	0.52	0.41	0.79	0.70
Group 12	0.50	0.61	0.32	0.70	0.71	0.52
Group 13	0.49	0.41	0.61	0.72	0.73	0.73
Group 14	0.33	0.51	0.50	0.54	0.71	0.70
Group 15	0.49	0.35	0.50	0.69	0.53	0.69
Group 16	0.48	0.33	0.63	0.68	0.61	0.86
Group 17	0.53	0.64	0.41	0.74	0.76	0.62
Group 18	0.51	0.52	0.39	0.73	0.75	0.70
Group 19	0.33	0.60	0.44	0.52	0.75	0.65
Group 20	0.54	0.37	0.42	0.75	0.63	0.62
**Average (60 participants)**	**0.47**			**0.66**		

## Data Availability

The datasets in this study are available on request to the corresponding author.
