# Corrigendum to “A Collaborative Brain-Computer Interface Framework for Enhancing Group Detection Performance of Dynamic Visual Targets”

**DOI:** 10.1155/2022/9819804

**Published:** 2022-10-26

**Authors:** Xiyu Song, Ying Zeng, Li Tong, Jun Shu, Qiang Yang, Jian Kou, Minghua Sun, Bin Yan

**Affiliations:** ^1^Henan Key Laboratory of Imaging and Intelligent Processing, PLA Strategic Support Force Information Engineering University, Zhengzhou, China; ^2^The Clinical Hospital of Chengdu Brain Science Institute, MOE Key Lab for Neuro Information, University of Electronic Science and Technology of China, Chengdu, China; ^3^Xi'an Satellite Control Center, Hangzhou, China; ^4^PLA 32317 Force, Wulumuqi, China; ^5^Department of Radiology, Henan Provincial People's Hospital, Department of Radiology, Central China Fuwai Hospital, Zhengzhou University, Zhengzhou, China

In the article titled “A Collaborative Brain-Computer Interface Framework for Enhancing Group Detection Performance of Dynamic Visual Targets” [1], Figure 8 has been adapted from the study by Valeriani and Poli [2], which was referenced in the article as reference 33 but the adaptation was unacknowledged.

The corrected figure legend for Figure 8 has been given as follows where the adaptation is acknowledged.

“Summary of different BCI frameworks: (a) sBCI framework; (b) SC-cBCI framework; (c) MC-cBCI framework; (d) MLDANet-cBCI framework (adapted from [33] under a CC-BY 4 license (https://creativecommons.org/licenses/by/4.0/)).”
